# Insights into the reason of Human-Residential Bifidobacteria (HRB) being the natural inhabitants of the human gut and their potential health-promoting benefits

**DOI:** 10.1093/femsre/fuaa010

**Published:** 2020-04-22

**Authors:** Chyn Boon Wong, Toshitaka Odamaki, Jin-zhong Xiao

**Affiliations:** Next Generation Science Institute, Morinaga Milk Industry Co., Ltd., 5-1-83, Higashihara, Zama, Kanagawa, 252–8583 Japan; Next Generation Science Institute, Morinaga Milk Industry Co., Ltd., 5-1-83, Higashihara, Zama, Kanagawa, 252–8583 Japan; Next Generation Science Institute, Morinaga Milk Industry Co., Ltd., 5-1-83, Higashihara, Zama, Kanagawa, 252–8583 Japan

**Keywords:** bifidobacteria, human health, natural inhabitants, physiological properties, genetic adaptation, ecological fitness

## Abstract

Members of *Bifidobacterium* are among the first microbes to colonise the human gut, and certain species are recognised as the natural resident of human gut microbiota. Their presence in the human gut has been associated with health-promoting benefits and reduced abundance of this genus is linked with several diseases. Bifidobacterial species are assumed to have coevolved with their hosts and include members that are naturally present in the human gut, thus recognised as Human-Residential Bifidobacteria (HRB). The physiological functions of these bacteria and the reasons why they occur in and how they adapt to the human gut are of immense significance. In this review, we provide an overview of the biology of bifidobacteria as members of the human gut microbiota and address factors that contribute to the preponderance of HRB in the human gut. We highlight some of the important genetic attributes and core physiological traits of these bacteria that may explain their adaptive advantages, ecological fitness, and competitiveness in the human gut. This review will help to widen our understanding of one of the most important human commensal bacteria and shed light on the practical consideration for selecting bifidobacterial strains as human probiotics.

## Teaser

Bifidobacterial species that ubiquitously encounter in the human host, collectively known as the Human-Residential Bifidobacteria (HRB), possess unique genetic elements and superior physiological functions that contribute to their ecological fitness and competitiveness, and they are thus more beneficial to humans.

## INTRODUCTION

Members of the genus *Bifidobacterium* are of substantial importance due to their purported health-promoting effects in human across their lifespan (O'Callaghan and van Sinderen 2016). Their presence in the human gastrointestinal tract is often associated with health benefits including the production of metabolites such as short-chain fatty acids and vitamins, immune system development and prevention of gut disorders (O'Callaghan and van Sinderen 2016). Recent advances in bifidobacterial research reveal that bifidobacterial strains have coevolved with their hosts and many physiological characteristics can be residential-origin dependent (Lamendella *et al*. 2008; Sun *et al*. 2015; Wong *et al*. 2018; Zou *et al*. 2019; Rodriguez and Martiny 2020). In this regard, some species of bifidobacteria have been identified as the natural inhabitants of the human host (Wong *et al*. 2018). Nevertheless, the reason why bifidobacterial species reside in the human gut across the life course, their adaptation and survival in the harsh environment of the human gastrointestinal tract and their impact on human health remain elusive. Thus, exploration of their human niche-specific adaptations and functional traits as members of human gut microbiota is of utmost significance.

This review aims at providing an overview of the ecological fitness of the bifidobacterial species that are commonly present in the human gut and elucidate how this bacterial group contribute to human health. Here we summarise current evidence on the colonisation, genetic adaptation and physiological characteristics of the human gut commensal bifidobacteria and address their roles as members of human gut microbiota. An increased understanding of the physiological attributes of this genus and their functions in the human host, which are of immense industrial value, will aid the selection of probiotic strains for human use.

## COLONISATION OF BIFIDOBACTERIA IN THE HUMAN GUT

*Bifidobacterium* is one of the most abundant bacterial genera present in the healthy infant gut (Favier *et al*. 2002; Odamaki *et al*. 2016). The abundance of this genus in the human gastrointestinal tract substantially decrease after weaning and continue to decrease with age (Yatsunenko *et al*. 2012; Kato *et al*. 2017). During adulthood the levels decrease considerably but remain relatively stable; decreasing again in old age (Odamaki *et al*. 2016). Colonisation by bifidobacteria is believed to play pivotal roles in maintaining human health. It is associated with a range of beneficial health effects, involved in the maturation of the immune, digestive and metabolic systems and consequently protecting against the susceptibility to various diseases later in life (Turroni *et al*. 2008).

Initial bifidobacterial colonisation occurs from birth and is influenced by several extrinsic factors (Arboleya *et al*. 2016). It has become evident that bifidobacterial species are vertically transmitted from the mother and colonise the intestine of the infant at the very early stages of life (Makino 2018). Several studies have linked the transmission of bifidobacteria from the mother's vaginal tract, gastrointestinal tract, breast milk, placenta and amniotic fluid to the infant (Makino *et al*. 2013; Milani *et al*. 2015b; Collado *et al*. 2016; Ferretti *et al*. 2018; de Goffau *et al*. 2019).

The delivery mode, in particular, has been demonstrated to potently impact this initial colonisation, with vaginally born infants displaying an increased abundance of bifidobacteria compared to those born by caesarean section (Rutayisire *et al*. 2016; Reyman *et al*. 2019). More specifically, a study based on analysis of the gut microbiota of mothers and corresponding children demonstrated that vaginally delivered infants share at least one monophyletic strain belonging to the genus *Bifidobacterium* with their mothers, whereas the monophyletic strains were not observed among infants delivered by caesarean section, which is thus indicative of vertical transmission (Makino *et al*. 2013). Moreover, in recent comparative genome analysis, the strains of bifidobacteria isolated from vaginal and gut microbiomes were indistinguishable, thus enforcing the importance of the maternal vaginal microbiome as a source of bifidobacterial colonisation (Freitas and Hill 2018).

The maternal birth canal has therefore been implicated as an essential source of bacteria, including bifidobacteria, during the delivery process. Although the colonisation of the gut environment was traditionally thought to begin at birth; new evidence of the presence of bacteria in the uterine environment suggests a primary foetal colonisation (de Goffau *et al*. 2019). Recent studies also suggest that many foetuses are exposed to microbes through the amniotic fluid that was continuously swallowed from mid to late gestation *in utero* (Chu *et al*. 2017). However, the ‘in utero colonisation hypothesis’ remains subject to debate. It is argued that, due to methodological difficulties, current scientific evidence does not support the existence of microbiome within the healthy foetal milieu (Perez-Muñoz *et al*. 2017). A recent study also demonstrates that there was no evidence for the presence of bacteria in the human placenta (de Goffau *et al*. 2019). Therefore, conclusions remain unachievable, and more studies are needed in this area.

Also, during vaginal delivery, as the infants pass through the birth canal, their oral and nasal cavities are infiltrated with vaginal secretions (Torres-Alipi *et al*. 1990; Li *et al*. 2018). For this reason, the neonatal oral fluid at delivery appears to be a mother-to-child-transmission route for bifidobacteria. It is indeed true that bifidobacteria are present in the neonatal oral fluid at birth and the same strains are detected in both the oral fluid and faecal samples collected at one month after birth, suggesting neonatal oral fluid at delivery is an essential source of bacteria from both maternal and environmental ecosystems and may contribute to the early delivery of bacteria to the digestive tracts of neonates (Toda *et al*. 2019).

Furthermore, accumulating evidence suggests that human breast milk harbours a microbial community and represents a source of commensal bacteria for the neonates (Gueimonde *et al*. 2007; Jost *et al*. 2014; Ruiz, García-Carral and Rodriguez 2019). It has been reported that bifidobacterial strains were present in maternal milk, suggesting the possibility of breast milk as a transmission route of bifidobacteria from the mother to the infant gut (Jost *et al*. 2014; Makino *et al*. 2015; Kordy *et al*. 2020). In this context, feeding mode is considered to be some of the major forces that could impact the colonisation level and species composition of bifidobacteria in the infant gut. Breastfeeding is known to provide the infant with a broad spectrum of biologically active factors including, among others, human milk oligosaccharides (HMOs), that aid in the colonisation of bifidobacteria, as well as a set of commensal microbes to initially colonise the infant gut (Le Doare *et al*. 2018). Numerous studies comparing the infant gut microbiome of exclusively breast-fed versus formula-fed infants have reported that the guts of breast-fed infants are enriched with *Bifidobacterium* spp. whereas formula-fed infants have a lower abundance of beneficial bacteria (Wang *et al*. 2015; Lewis and Mills 2017).

Altogether, these studies provide clues of where and how bifidobacteria colonise the human gut. It is implicated that early life colonisation patterns and successions of bifidobacteria may contribute to the risk of developing health complications during neonatal stage or later in life, highlighting the importance of maintaining proper levels of bifidobacteria in the human gut (Arboleya *et al*. 2016).

## GENERAL FEATURES OF *BIFIDOBACTERIUM* SPECIES

Bifidobacteria are Gram-positive, anaerobic, non-motile, non-spore-forming, polymorphic rods that belong to the family *Bifidobacteriaceae*, order *Bifidobacteriales* and phylum Actinobacteria. Bifidobacteria display a range of distinct cell forms, including curved, short and bifurcated Y shapes. The genomic DNA of bifidobacteria contains a high guanine-plus-cytosine content, with numerous genes involved in the metabolism of dietary and host-derived carbohydrates (Milani *et al*. 2014; Ventura *et al*. 2014). At present, the genus *Bifidobacterium* encompasses approximately 80 species, including four species (*Bifidobacterium animalis*, *B. longum*, *B. pseudolongum* and *B. thermacidophilum*) that are further divided into subspecies (Parte 2018; Sakanaka *et al*. 2020).

In addition to the presence in the human gut, bifidobacterial species also naturally occur in the gastrointestinal tract of animals as well as a few that are present in sewage, human vagina, oral cavity, breast milk, and foods (Ventura *et al*. 2007). Bifidobacteria are widely distributed in the gut of social animals (e.g. mammals, birds and insects), whose offspring are dependent on parental care (Turroni, Van Sinderen and Ventura 2011). Vertical transmission from mother to offspring appears to be a common ecological trademark of this bacterial genus, reflecting a clear evolutionary link between the parent, offspring/progeny and the bifidobacterial species present. A study has revealed that the clades of *Bifidobacteriaceae* arose via cospeciation with humans, chimpanzees, bonobos and gorillas over the past 15 million years, among which the species have been maintained exclusively within host lineages across hundreds of host generations (Moeller *et al*. 2016). After that, bifidobacteria have developed a diverse number of genetic strategies to adapt to their respective hosts and display differences in their ecological adaptation among species.

**Figure 1.**
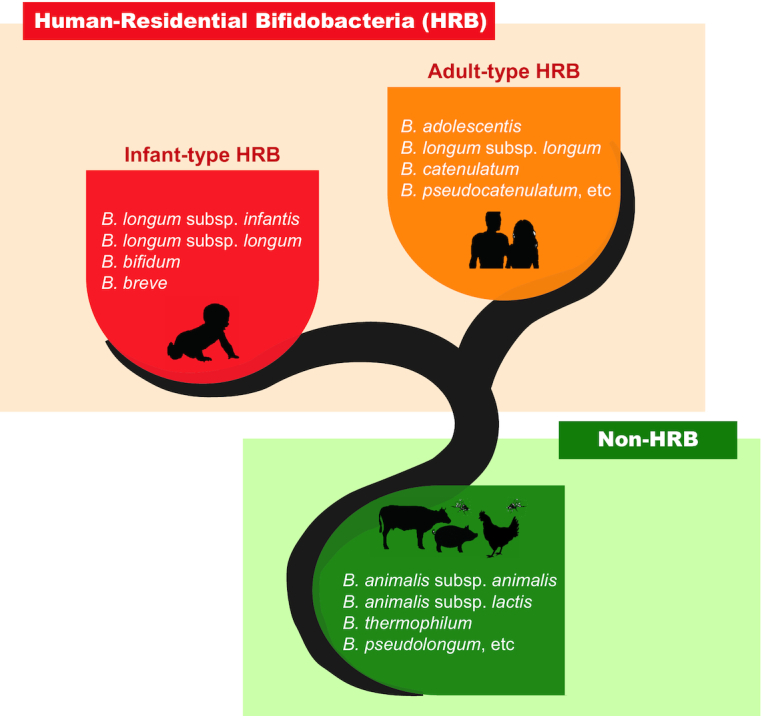
Distinctive differences in ecological distribution of bifidobacteria. Bifidobacterial species are distributed in a wide range of niches, encompassing the human intestine, the gastrointestinal tract of animals, human vagina, human oral cavity, breast milk, sewage and food. The species that naturally occur in the human host are referred to as Human-Residential Bifidobacteria (HRB). Among HRB, *Bifidobacterium longum* subsp. *infantis*, *B. longum* subsp. *longum*, *B. bifidum* and *B. breve* are recognised as the four exclusive members of the infant gut and are referred to as infant-type HRB. Meanwhile, bifidobacterial species that are predominantly present in the adult gut are referred to as adult-type HRB. Conversely, other species which are the natural inhabitants of animals or environment are referred to as non-HRB. The species of HRB and non-HRB display differences in their ecological adaptation.

In general, bifidobacteria could be categorised into two major groups based on their residential origins; bifidobacterial species that are naturally encountered in the human gastrointestinal tract are referred to as Human-Residential Bifidobacteria (HRB), whereas other species which are the natural inhabitants of animals or environment as non-HRB (Sugahara, Odamaki and Xiao 2015; Wong *et al*. 2018) (Fig. 1). Among HRB, *B. breve, B. longum* subsp. *infantis, B. longum* subsp. *longum* and *B. bifidum*, which are the dominant species in the infant's intestines, are referred to as infant-type HRB (Turroni *et al*. 2012), whereas *B. adolescentis*, *B. catenulatum*, *B. pseudocatenulatum*, *B. longum* subsp. *longum*, etc., which are the dominant species in the adult intestines, are referred to as adult-type HRB (Ishikawa *et al*. 2013). However, there does not seem to be an absolute infant versus adult division of bifidobacterial (sub)species (Turroni *et al*. 2012, 2019). It is noted that HRB strains might not be adapted to particular habitats (gut/blood/human milk) and life stages (infant/adult) within humans, for which the phylogenetic and genomic traits are undisguisable from the strains studied (Freitas and Hill 2018; Rodriguez and Martiny 2020). After that, *B. longum* subsp. *longum* was referred to as species that predominantly inhabit both the infant and adult intestines (Odamaki *et al*. 2018). The ubiquitous distribution of *B. longum* subsp. *longum* species across the human lifespan was suggested to be associated with their genetic diversity which could enhance their adaptation and increase competitiveness in the gut environment, and at least partly due to extensive transmission across family members, a phenomenon that was shown not to be confined to mother-infant pairs (Odamaki et al. 2018, 2019).

Meanwhile, non-HRB species encompass *B. animalis* subsp. *animalis*, *B. animalis* subsp. *lactis*, *B. thermophilum*, *B. pseudolongum*, etc., among which some species show a strict ecological adaptation to a particular animal gut (Lamendella *et al*. 2008). For instance, the non-HRB species *B. magnum* and *B. cuniculi* were ubiquitously found in rabbit faeces, *B. pullorum* and *B. gallinarum* in the chicken intestine, and *B. longum* subsp. *suis* in the piglet faeces (Biavati *et al*. 2000). Such variations were suggested to be related to the diet, age, and species of the host animals (Mitsuoka and Kaneuchi 1977). Bifidobacteria are also found in other atypical ecological niches that are either partly linked with the gut: sewage (e.g. *B. minimum* and *B. thermacidophilum*) (Biavati, Scardovi and Moore 1982; Dong *et al*. 2000) or completely different from that of the gut: food products (e.g. *B. mongoliense* from fermented mare's milk product and *B. aquikefiri* and *B. tibiigranuli* from water kefir) (Watanabe *et al*. 2009; Laureys *et al*. 2016; Eckel *et al*. 2019). Notably, a small population of *B. animalis* subsp. *lactis*, which is originated from animal gut and commonly incorporated as probiotics in dairy products, was also detected in human faeces (Turroni *et al*. 2009). It has been suggested that most of the strains of *B. animalis* subsp. *lactis* currently applied in commercial products were genetically indistinguishable (Xiao *et al*. 2010b; Milani *et al*. 2013). Its detection might attribute to human diets, such as intake of yoghurt, suggesting that *B. animalis* subsp. *lactis* is not a commensal of human gut microbiota (Kato *et al*. 2017). Altogether, the identification of bifidobacteria in these environments could be plausibly a consequence of ‘natural’ contaminations from human/animal gut origins and/or from accidental contaminations during the sampling procedures. Conversely, there could be a strong rationale behind the species of HRB being the natural inhabitant of the human gut. As will be discussed later, accumulating evidence suggests that the residential origins of bifidobacteria are associated with a number of significant differences in their physiological features and health-promoting functions in the human host (Xiao *et al*. 2010a; Odamaki *et al*. 2015; Sugahara *et al*. 2015; Sugahara, Odamaki and Xiao 2015; Minami *et al*. 2016; Sakurai *et al*. 2017, 2018; Wong *et al*. 2018, 2020; Sakurai, Odamaki and Xiao 2019).

## NATURAL SELECTION OF INFANT-TYPE HRB BY HUMAN MILK

**Figure 2.**
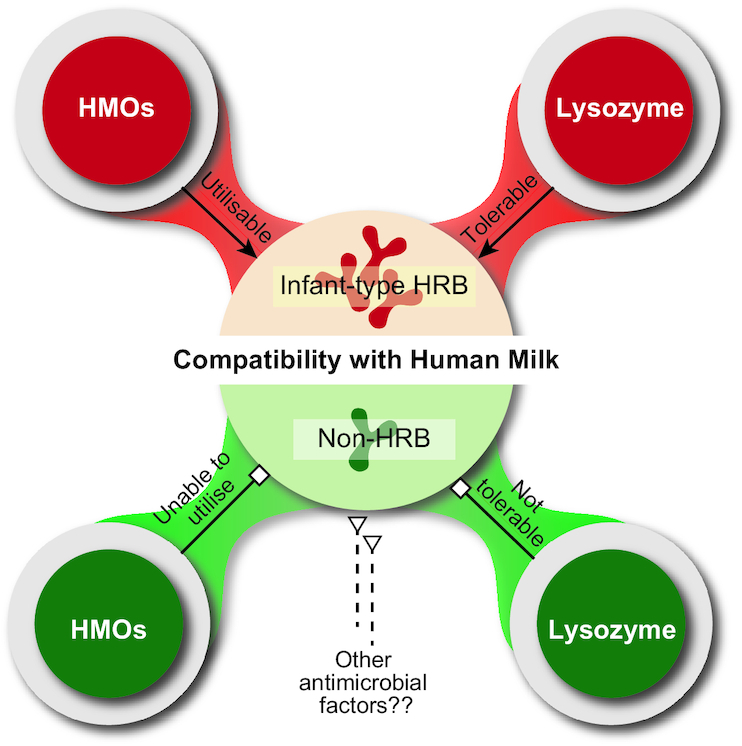
Differences in the compatibility with human milk among bifidobacterial species. Infant-type HRB species are adapted to utilise human milk oligosaccharides (HMOs), which are one of the most important component in the human milk that function to selectively support the colonisation of bifidobacteria in infant gut. Infant-type HRB are also highly tolerant to the antibacterial component present in the human milk called lysozyme. In contrary, non-HRB species are excluded by these selective factors present in the human milk. The species of non-HRB are lacking with the enzymatic arsenal dedicated for HMOs utilisation and are sensitive to human milk lysozyme.

Human milk is regarded as the principal source of nutrition for infants. It contains a rich source of essential nutrients that nourishes the neonate, supporting proper growth and development. In addition, human milk also provides the neonate with its own microbiota as well as a wide array of bioactive molecules that indirectly can contribute to the establishment of infant gut microbiome (Le Doare *et al*. 2018). Breast-fed infants are characterised by a gut population dominated by the species of HRB, including *B. bifidum*, *B. breve*, *B. longum* subsp. *longum*, and *B. longum* subsp. *infantis* (Turroni *et al*. 2012; Makino *et al*. 2015). This predominance has been explained in part by the high amounts of a class of molecules called human milk oligosaccharides (HMOs) (Lawson *et al*. 2019). Intriguingly, these molecules are indigestible by the infant, and thus provide no direct nutritive value. Instead, HMOs are thought to selectively support the colonisation of HRB species capable of utilising these diverse substrates (Sela and Mills 2010; Asakuma *et al*. 2011; Katayama 2016). In addition to HMOs, human milk contains antibacterial compounds such as immunoglobulins, lactoferrin, lactoferricin, lysozyme, lactoperoxidase, free fatty acids, antimicrobial peptides and others (Field 2005). Among them, lysozyme has been reported to be another possible selective factor in human milk that could affect the colonisation of bifidobacterial species in the infant gut (Gagnon *et al*. 2004; Rada *et al*. 2010; Minami *et al*. 2016) (Fig 2).

### HMO utilisation

HMOs are the third most abundant substantial component in human milk after lactose and lipids, reaching concentrations of up to 15 g/L in mature human milk (Kobata 2010). HMOs are complex carbohydrates composing of five monosaccharides: glucose (Glc), galactose (Gal), *N*-acetylglucosamine (GlcNAc), fucose (Fuc) and *N*-acetylneuraminic acid (NeuAc) or sialic acid (Kirmiz *et al*. 2018). HMOs are synthesised from a lactose (Galβ1–4Glc) backbone at the reducing end and are elongated by the repeats of *β*-1,3- or *β*-1,6-linked lacto-N-biose I (LNB) or *N*-acetyllactosamine (Chen 2015). The terminal lactose molecules can be further decorated with the addition of fucose and/or sialic acid residues (Bode and Jantscher-Krenn 2012). With varying degrees of polymerisation and multiple linkage isomers, more than 200 different structures of HMOs have been observed (Thomson, Medina and Garrido 2018). The overabundant examples of HMOs are lacto-*N*-tetraose (LNT), lacto-*N*-fucopentaose I (LNFP I), lacto-*N*-difucohexaose I (LNDFH I) and 2’-fucosyllactose (2’-FL), with three of the four HMOs are type I chains that exhibit the core structure of LNT (Kunz *et al*. 2000; Chen 2015). Degradation of LNT and its constituent component, LNB, has therefore been implicated as an essential step in HMO metabolism.

#### Utilisation of HMOs by bifidobacteria

Bifidobacterial species display differential ability to consume various HMOs, among which the infant-type HRB, namely, *B. bifidum*, *B. breve*, *B. longum* subsp. *infantis* and *B. longum* subsp. *longum* are adapted to utilise HMOs, while the ability was not present in many species of adult-type HRB and the non-HRB (LoCascio *et al*. 2007; Xiao *et al*. 2010a; Asakuma *et al*. 2011). More specifically, considerable differences in HMO utilisation among the four species of infant-type HRB were observed. *B. longum* subsp. *infantis* aggressively consumes LNB and almost all types of HMOs including fucosylated and sialylated molecules, and the consumption capability is highly and widely conserved in this subspecies (Xiao *et al*. 2010a; Asakuma *et al*. 2011; Garrido *et al*. 2015; Thomson, Medina and Garrido 2018). Meanwhile, the capability to consume HMOs is somewhat variable in *B. bifidum* strains, utilising almost all classes of HMOs and LNB (Xiao *et al*. 2010a; Asakuma *et al*. 2011; Garrido *et al*. 2015; Gotoh *et al*. 2018; Thomson, Medina and Garrido 2018). By contrast, the ability of the *B. breve* and *B. longum* subsp. *longum* strains to assimilate HMOs is limited. Most of the *B. breve* strains can utilise only LNT, lacto-*N*-neotetraose (LNnT), and LNB (Xiao *et al*. 2010a; Asakuma *et al*. 2011; Ruiz-Moyano *et al*. 2013; James *et al*. 2016; Thomson, Medina and Garrido 2018), whereas the majority of the *B. longum* subsp. *longum* strains can consume solely LNT and LNB, leaving other HMOs unmodified (Xiao *et al*. 2010a; Asakuma *et al*. 2011; Garrido *et al*. 2016; Thomson, Medina and Garrido 2018). Moreover, a few strains of these two (sub)species have been described to be capable of assimilating exceptionally other complex fucosylated HMOs, such as 2’-FL, 3-fucosyllactose (3-FL), lactodifucotetraose (LDFT) and LNFP I/II/III (Ruiz-Moyano *et al*. 2013; Garrido *et al*. 2016; Matsuki *et al*. 2016).

In addition, HMO utilisation capability by other strains of HRB (*B. kashiwanohense* and *B. pseudocatenulatum*) has been reported (Xiao *et al*. 2010a; Bunesova, Lacroix and Schwab 2016). *B. kashiwanohense* was found to consume preferably 2’-FL and 3-FL rather than LNT and LNnT (Bunesova, Lacroix and Schwab 2016; James *et al*. 2019). *B. pseudocatenulatum* was shown to be capable of utilising LNT (Matsuki *et al*. 2016). The ability to consume 2’-FL, 3-FL, LDFT and LNB has also been described in some strains of *B. pseudocatenulatum* (Xiao *et al*. 2010a; Matsuki *et al*. 2016). Nonetheless, the majority of adult-type HRB and non-HRB are unable to utilise HMOs. It is not surprising that such capability is absent and/or has been lost in some adult-type HRB and non-HRB that typically reside in the adult and animal intestines as well as other ecological niches.

#### Strategies in utilisation of HMOs by infant-type HRB

Infant-type HRB species have evolved two different strategies to degrade HMOs (LoCascio *et al*. 2009; Garrido, Barile and Mills 2012; Katayama 2016). The first strategy is oligosaccharide transporter-dependent, as observed in *B. longum* subsp. *infantis*, *B. breve* and most strains of *B. longum* subsp. *longum*, where a wider array of ATP-binding cassette (ABC) transporters (solute-binding proteins [SBPs]) are required for consumption of a broader set of HMOs; while the second is extracellular glycosidase-dependent, as observed in *B. bifidum* and certain strains of *B. longum* subsp. *longum* that contain the gene encoding lacto-*N*-biosidase (LnbX), where fewer transporters but cell wall-associated glycosyl hydrolases (GHs) are required for HMO utilisation. In the former case, intact HMOs are directly imported into the cells by ABC transporters where they are then hydrolysed intracellularly for consumption. In the latter case, HMOs are hydrolysed outside the cells by cell wall-associated GHs to liberate mono- and/or disaccharides. These liberated carbohydrates are subsequently catabolised inside the cells after their internalisation. This extracellular glycosidase-dependent strategy could then lead to cross-feeding of HMO degradants within *Bifidobacterium* community whereby the extracellularly liberated mono- and disaccharides may be consumed by other bifidobacterial species that are not able to consume the more complex HMOs (Gotoh *et al*. 2018). This has been observed in the case of sialyllactose-mediated cross-feeding between *B. bifidum* and *B. breve* (Egan *et al*. 2014b; Nishiyama *et al*. 2018). In these cases, *B. breve*, which is a non-sialyllactose consumer, can grow on the residual sialic acid that is liberated from sialyllactose by the extracellular sialidase of *B. bifidum*. Noteworthy, given that the abundance of extracellular glycosidase-dependent *B. bifidum* and LnbX-positive *B. longum* subsp. *longum* is generally lower in the infant gut microbiota (Matsuki *et al*. 2016; Yamada *et al*. 2017), it is suggested that most of the infant-type HRB employ a transporter-dependent strategy for HMO assimilation.

Comparative genomic analysis has exemplified the functional capabilities of infant-type HRB to assimilate HMOs. Infant-type HRB, including *B. breve*, *B. bifidum*, *B. longum* subsp. *longum*, and *B. longum* subsp. *infantis*, are equipped with sets of HMO-related genes, and such genetic elements are hardly detected in the genomes of the majority species of adult-type HRB and the non-HRB (Garrido *et al*. 2015; Odamaki *et al*. 2015; James *et al*. 2016, 2019; Sakanaka *et al*. 2020). The conservation and prevalence of the HMO utilisation genes were remarkably variable among infant-type HRB species. Infant-type HRB species employ different HMO metabolic pathways with different degrees of degradation and size limitations. It appears that HMO-related genes are highly conserved in the genome of *B. longum* subsp. *infantis* for which it contains an army of intracellular GHs and ABC transporters that are necessary for HMO utilisation (Sela *et al*. 2008; LoCascio *et al*. 2010). Notably, *B. longum* subsp. *infantis* internalises particular, intact small-mass HMOs, relying on the ABC transporters with defined specificity for individual HMO families (Garrido *et al*. 2011). In contrast, *B. bifidum* relies on a set of diverse cell wall-associated extracellular GHs including lacto-*N*-biosidase (LnbB) and 1,2-α-L-fucosidase that exhibit similar enzymatic affinities for HMOs compared to the intracellular enzymes from *B. longum* subsp. *infantis* (Wada *et al*. 2008; Ashida *et al*. 2009; Turroni *et al*. 2010; Kitaoka 2012; Sela *et al*. 2012). All genes required for extracellular HMO degradation are highly conserved across the species of *B. bifidum* (Gotoh *et al*. 2018; Sakanaka *et al*. 2020).

On the other hand, the species of *B. breve* shows high conservation of genes encoding for the SBP of ABC transporters (LNnT-BP (NahS) and GNB/LNB-BP (GltA)) and intracellular enzymes (GH 42 LNT *β*-1,3-galactosidase, GH 20 *β*-*N*-acetylglucosaminidase, GH 2 *β*-1,4-galactosidase and GH 112 GNB/LNB phosphorylase) that are necessary for the degradation of LNT, LNnT and LNB (James *et al*. 2016; Sakanaka *et al*. 2020). It was found that, although this species commonly possesses an intracellular GH95 α-fucosidase, the prevalence of the fucosyllactose transporters (FL1-BP and FL2-BP) is remarkably low (Sakanaka *et al*. 2019), and thus exhibit strain-dependent differences in the capability to utilise fucosylated HMO. Indeed, only a few strains of *B. breve* have been shown to contain GH29 α-fucosidase and the ABC transporter system and are capable of assimilating 2’-FL and larger fucosylated HMOs such as LNFP I/II (Ruiz-Moyano *et al*. 2013; Matsuki *et al*. 2016). Moreover, it has been described that the strain *B. breve* UCC2003 utilises LNT and LNnT through two different mechanisms, which resemble the degradation pathway of *B. longum* subsp. *infantis* (James *et al*. 2016). *B. breve* UCC2003 was also shown to possess functional pathways to internalise and utilise L-fucose (James *et al*. 2019).

The strains of *B. longum* subsp. *longum* who are recognised as LNT/LNB consumers possess the SBP of ABC transporter (GNB/LNB-BP (GltA)) as well as LNT- and LNB-degrading intracellular enzymes including GH42 LNT *β*-1,3-galactosidase, GH20 *β*-*N*-acetylglucosaminidase, GH2 *β*-1,4-galactosidase and GH 112 GNB/LNB phosphorylase (Suzuki *et al*. 2008; Garrido *et al*. 2016; Sakanaka *et al*. 2020). Moreover, certain strains of *B. longum* subsp. *longum* also contain extracellular GH136 lacto-*N*-biosidase (LnbX) enzyme in its genome (Sakurama *et al*. 2013). Noteworthy, strains containing LnbX mainly degrade LNT to LNB and lactose, and subsequently import LNB with the SBP of ABC transporters (GNB/LNB-BP (GltA)) and use GNB/LNB phosphorylase (LnpA) for intracellular phosphorolysis, whereas LnbX-negative strains hydrolyse LNT inside the cell with the enzymes LNT β-1,3-galactosidase, β-*N*-acetylglucosaminidase, and β-1,4-galactosidase (Sakurama *et al*. 2013; Odamaki *et al*. 2015). Recent studies have also found that specific strains of *B. longum* subsp. *longum* are able to utilise 2’-FL and 3-FL as the unique carbon sources (Garrido *et al*. 2016; Arboleya *et al*. 2018). These strains were found to possess a novel gene cluster devoted to the utilisation of fucosylated HMOs, including genes encoding an α-fucosidase enzyme, the ABC transporter system that is responsible for import of fucosylated molecules, and components for fucose metabolism (Garrido *et al*. 2016; Arboleya *et al*. 2018).

Interestingly, a recent report has highlighted that some strains of HRB could employ a unique adaptation strategy to assimilate fucosylated HMOs (Sakanaka *et al*. 2019). The study identified two functionally distinct but overlapping fucosyllactose transporters (FL transporter-1 and -2) that are highly conserved in *B. longum* subsp. *infantis*. FL transporter-2 is capable of taking up 2’-FL, 3-FL, LDFT, and LNFP I, whereas FL transporter-1 can import only the former two molecules. The distinct specificities between FL transporter-1 and -2 (the homologous ABC transporters) may be determined by the partially different FL-binding sites of the corresponding SBPs (Sakanaka *et al*. 2019). The homologs of FL transporter-1 or -2 are also sporadically distributed among the infant-type HRB species (*B. longum* subsp. *longum* and *B. breve*) as well as a few strains of *B. pseudocatenulatum*, *B. kashiwanohense* and *B. longum* subsp. *suis* (James *et al*. 2019). It appears that the different capabilities of bifidobacteria to assimilate the fucosylated HMOs are mostly attributed to conservation profiles of FL transporter-1 and/or -2 in bifidobacteria, emphasising the pivotal role of these transporters in dictating the preference of HMO uptake (Garrido *et al*. 2016; Matsuki *et al*. 2016; Sakanaka *et al*. 2019). Furthermore, the SBP homolog gene of FL transporter-2 was found to be positively and actively associated with the abundance of *Bifidobacterium* in the gut of breast-fed infants, suggesting the FL transporter-2 may function as a pivotal fitness factor involved in the dominance and adaptation of bifidobacterial species, particularly infant-type HRB, in the infant gut microbiota (Sakanaka *et al*. 2019). Another recent report also reinforced the importance of fucosylated HMO metabolism for the successful bifidobacterial establishment in the breast-fed infant gut (James *et al*. 2019). The study revealed that the four gene homologs (FumFDCE) involved in fucose/fucosylated HMO utilisation pathway are distributed in *Bifidobacterium* strains typically found in infants, including *B. breve* and *B. longum* subsp. *infantis* (James *et al*. 2019). Collectively, these findings provide intriguing insights into the regulatory networks behind HMO utilisation. It is implicated that infant-type HRB has undergone specific genetic adaptation to and strict co-evolution with the infant host. In addition to the implications to host-bacterial coevolution, this wide array of responses to HMOs may impact probiotics design and applications, where the residential origin and strain-level differences in substrate utilisation require additional considerations.

### Tolerance to lysozyme

A recent study has demonstrated that bifidobacterial species of different residential origins exhibit differential growth characteristics in human milk (Minami *et al*. 2016). The ability to grow in human milk was found to be highly conserved in infant-type HRB strains of *B. longum* subsp. *infantis* and *B. breve*, while the growth characteristics of *B. longum* subsp. *longum* and *B. bifidum* were shown to be strain-dependent; some strains grew and some retained their inoculated cell numbers. In particular, the tested strains of adult-type HRB and non-HRB were unable to grow in human milk and had reduced viable cell numbers after incubation. Given the fact that human milk also contains an essential amount of lactose (approximately 7% by weight) (Ballard and Morrow 2013), a carbohydrate source that can be assimilated by bifidobacteria, it seems likely and has indeed been shown to be true that nutrient deficiency may not be the reason for the repressed growth and that the presence of lysozyme in human milk is related to the inability of the strains of adult-type HRB and non-HRB to grow in human milk (Minami *et al*. 2016).

Lysozyme is a ubiquitous antibacterial enzyme that is present in large quantities in human milk (up to 400 µg/mL), which is approximately 1000–3000-fold higher concentration than the bovine milk (Prieur 1986). In addition to human milk, lysozyme is naturally present in almost all tissues and biological fluids, such as tears, saliva, sweat, and mucus (Field 2005). It is therefore plausible that lysozyme susceptibility might represent a threat for bifidobacteria to survive in the human body. Studies have shown that bifidobacterial species that are the natural inhabitants of human intestines (HRB) are more resistant to lysozyme compared to those that are commonly encountered in the animal intestines (non-HRB) (Rada *et al*. 2010; Rockova *et al*. 2012; Minami *et al*. 2016). More specifically, infant-type HRB strains were tolerant to high concentrations of lysozyme, and lysozyme tolerance was positively correlated with their growth in human milk. In contrast, the tested strains of adult-type HRB displayed intermediate tolerance, while non-HRB were relatively sensitive to lysozyme, which would, in turn, led to their failure to grow in human milk (Minami *et al*. 2016). One study examining the tolerance mechanisms of HRB strains against lysozyme has revealed that differences of lysozyme susceptibility between HRB and non-HRB are independent of the peptidoglycan-degrading property of lysozyme (Sakurai *et al*. 2017). Instead, the tolerance to lysozyme among some HRB strains is due to their resistance to the cationic properties of lysozyme that are associated with the cytotoxic activity (Sakurai *et al*. 2017). This finding provides a fundamental insight into how HRB are protected against lysozyme action, which would, in turn, contribute to their colonisation in the human host. Further research is needed to elucidate the specific mechanisms in HRB against lysozyme hydrolysis. Altogether, these findings suggest that the presence of lysozyme in human milk could act as another crucial selective factor that prevents the colonisation of non-HRB in the guts of breast-fed infants.

### Bifidobacterial human milk compatibility and colonisation of infants

As aforementioned, bifidobacteria of different residential origins display genotypic and phenotypic variations in their compatibility with human milk. Importantly, not all bifidobacteria are equally adapted to consume HMOs and tolerant to human milk lysozyme, which suggests that certain strains of bifidobacteria (infant-type HRB) are better able to colonise a breast-fed infant. This concept has been addressed in a clinical study investigating the gut microbial composition of premature infants — infants who often have a delayed bifidobacterial colonisation in comparison to term infants (Underwood *et al*. 2013). Premature infants receiving milk were given the probiotic *B. longum* subsp. *infantis* and *B. animalis* subsp. *lactis*. The results demonstrated that *B. longum* subsp. *infantis*, a member of infant-type HRB, colonised better than *B. animalis* subsp. *lactis*, a non-HRB strain, in premature infants exposed to both formula and human milk feeding. This study suggests a close linkage between HMOs and human milk lysozyme (in this case, as part of whole human milk) and colonisation of the gut, specific to certain strains of (infant-type) HRB but not non-HRB (Underwood *et al*. 2013; Minami *et al*. 2016; Lawson *et al*. 2019).

Furthermore, HRB species (*B. longum* subsp. *longum*, *B. breve* and *B. bifidum*) but not the non-HRB species (*B. animalis*) were more frequently detected in the gut microbiota of breast-fed infants (Tannock *et al*. 2013). In contrast, non-HRB species were more abundant in formula-fed infants than in breast-fed infants, indicating the presence of HMOs and lysozyme in the human milk, but not the infant formulas, contributed to the different abundancy of bifidobacterial species in infant gut microbiota (Tannock *et al*. 2013; Minami *et al*. 2016). Another study evaluating the early gut microbial composition of healthy neonates also revealed that the colonisation of the non-HRB species, *B. animalis* subsp. *lactis*, was dictated by the type of feeding (Martin *et al*. 2016). Noteworthy, *B. animalis* subsp. *lactis* was more frequently detected in the gut microbiota of formula-fed infants and its colonisation was relatively weak in breast-fed infants, suggesting that *B. animalis* subsp. *lactis* might not be a common member of the infant gut microbiota. In contrast, the species of infant-type HRB such as *B. breve* or *B. longum* subsp. *infantis* were confirmed as early infant gut colonisers and their colonisation was not affected by the mode of delivery and type of feeding (Martin *et al*. 2016). Taken together, these data clearly demonstrated the superiority of infant-type HRB – bifidobacterial species of high compatibility with human milk – to colonise the infant gut, highlighting that certain infant-type HRB strains are more beneficial and could be a better probiotic candidate for human use, especially in infants. In this regard, there must be a good reason why human milk naturally selected the species of HRB, particularly the infant-type HRB, for the developing infants. It is implicated that HRB are better adapted to the intestinal environment of the human host and could contribute to better human health.

## PHYSIOLOGICAL FUNCTIONS OF HUMAN-RESIDENTIAL BIFIDOBACTERIA (HRB)

### Carbohydrate metabolism

Comparative genomic studies of bifidobacterial species have uncovered specific genetic strategies of the members of bifidobacteria to establish and persevere in the human gastrointestinal tract (Milani *et al*. 2014, 2015a; Odamaki *et al*. 2015). It appears that currently known bifidobacterial taxa have undergone a substantial number of gene acquisition events during the evolutionary process where such acquired genes – a specific genetic repertoire that encodes glycosyl hydrolases (GHs) – are related to carbohydrate metabolism. After that, adaptation to habitats rich in complex carbohydrates, as such in the human gut, is seen as the main driving force responsible for speciation among members of the genus *Bifidobacterium* (Milani *et al*. 2016).

Even though bifidobacteria are not a dominant member of the adult gut microbiota, their functional biological roles in the metabolism of dietary and host-derived glycans cannot be neglected. Bifidobacteria display saccharolytic behaviour and their ability to colonise and survive in the gastrointestinal tract is largely dependent on their carbohydrate metabolic capabilities (Milani *et al*. 2016). According to the Carbohydrate Active Enzymes (CAZy) classification, the pan-genome of the *Bifidobacterium* genus is among the largest predicted glycobiomes of the known gut commensals, with a large proportion of annotated genes encode an enzymatic arsenal involved in carbohydrate metabolism, including GHs, glycosyltransferases, and carbohydrate esterases (Milani *et al*. 2015a). The bifidobacterial glycobiome is enriched in enzymes belonging to the GH13 family, which are the typical enzymes for degradation of a wide range of complex carbohydrates, such as starch, glycogen, and related substrates (e.g. amylose, amylopectin, pullulan, maltodextrin and cyclomaltodextrin), as well as palatinose, stachyose, raffinose and melibiose (Milani *et al*. 2015a). Notably, all these sugars are dominant glycans found in the adult mammalian diet. Moreover, part of the predicted glycobiome of bifidobacteria is extracellular, allowing them to degrade carbohydrates whose direct uptake is not possible due to the size and complexity of the glycan.

Intriguingly, it appears that the occurrence of HRB in the human gut is supported by their metabolic abilities about various complex, host-indigestible carbohydrates either (in)directly derived from the host (i.e. mucin and HMOs) or the diet (Milani *et al*. 2014, 2015a, 2016). Analyses of the genome sequences of the various type strains representing HRB and non-HRB revealed an abundant presence of genes related to plant-derived carbohydrate metabolism in the genomes of HRB, especially the adult-type HRB, *B. adolescentis* and *B. longum* subsp. *longum*. The genome of *B. adolescentis* is particularly enriched with a much larger set of GH 13 enzymes, which include amylase, pullulanase, and cyclomaltodextrinase, than other HRB species, exemplifying their capabilities to metabolise various dietary starch and starch-like oligo/polysaccharides such as amylopectin, pullulan, maltotriose, and maltodextrin (Duranti *et al*. 2014, 2016). Such genetic architecture may reflect an adaptation of *B. adolescentis* species to the adult diet, which often contains high amounts of plant-derived glycans, and thus highlighting the superiority of this HRB species to persist in the gastrointestinal tract of adult human beings. Moreover, the analyses of fermentation profiles of *B. adolescentis* strains have further exemplified their preference for the utilisation of different sugars (e.g. galactose, mannose and glucose), as well as plant-derived carbohydrates that are typically present in the human diet, such as starch (Duranti *et al*. 2014).

Another important sign of bifidobacterial adaptation to the human gut is the ability of the HRB species, *B. longum* subsp. *longum*, to utilise a wide variety of plant-derived dietary carbohydrates. *B. longum* subsp. *longum* was shown to be genetically well equipped with a substantial number of GHs (e.g. xylanase, arabinosidase, galactosidase, neopullanase isomaltase, inulinase, glucosidase, hexosaminidase, and mannosidase), oligosaccharide transporters and proteins with a cell-surface anchor motif, reflecting the genetic strategies employed by this species to survive and compete in its ecological niche (Schell *et al*. 2002; Odamaki *et al*. 2015; Fujita *et al*. 2019). Noteworthy, a large number of GH 43 and 51 family members, which are enzymes responsible for the breakdown of arabinofuranoside and xylan, were found to be specific to *B. longum* subsp. *longum* as well as *B. adolescentis* and other adult-type HRB (Odamaki *et al*. 2015; Duranti *et al*. 2016; Komeno *et al*. 2019). The presence of such genetic elements for utilisation of plant-derived carbohydrates, which are assumed to be not introduced into infant gut before weaning, provides an advantage for this species to colonise in the human intestine. It appears that *B. longum* subsp. *longum* might play a key role in infant's digestion during weaning where a non-milk diet containing complex carbohydrates are being introduced for the first time. Consequently, such genetic architecture may explain why only *B. longum* subsp. *longum* predominantly inhabit in both the infant and adult intestines (Pacheco *et al*. 2015; Odamaki *et al*. 2018).

Moreover, *B. breve* is another HRB species that deserves a special mention as it appears to possess the ability to metabolise a wide range of *α*/*β*-glucose- and *α*/*β*-galactose-containing carbohydrates, corroborating its niche-specific adaptation to both infant and adult guts (Pokusaeva, Fitzgerald and van Sinderen 2011; Bottacini *et al*. 2018). Specific strains of *B. breve* encode various carbohydrate-modifying enzymes including α-glucosidases (belonging to the GH13 and GH31 families) that are involved in hydrolysis of *α*-glucosidic linkages usually present in di-, oligo- and polysaccharides (e.g. maltose, starch and related α-glycans) (Pokusaeva *et al*. 2009; Kelly *et al*. 2016), *β*-glucosidases (belonging to the GH1 and GH3 families) that are involved in assimilation of a variety of glycan substrates such as cellobiose and cellodextrin (Pokusaeva *et al*. 2011; Bottacini *et al*. 2018) and *β*-galactosidases (belonging to the GH2 and GH42 families) that are involved in degradation of lactose, galactan and galacto-oligossacharides (O'Connell Motherway, Fitzgerald and van Sinderen 2011; O'Connell Motherway *et al*. 2013). It has been found that certain *B. breve* strains were able to utilise starch, pullulan and amylopectin (Ryan, Fitzgerald and van Sinderen 2006). In addition, the strain *B. breve* UCC2003 is reported to encode various carbohydrate-modifying enzymes including amylopullulanase, endogalactanase, β-fructofuranosidase, β-1,4-glucosidase, α-1,6-glucosidases, and ribokinase) that allow it to metabolise various glycans such as sucrose, fructose, starch, amylopectin, panose, cellobiose, and ribose (Ryan, Fitzgerald and van Sinderen 2005; Motherway *et al*. 2008; O'Connell Motherway *et al*. 2009; Pokusaeva *et al*. 2010; O'Connell Motherway, Fitzgerald and van Sinderen 2011; Pokusaeva, Fitzgerald and van Sinderen 2011; Kelly *et al*. 2016). Given that *B. breve* is particularly abundant in early life where starch-containing foods, fruits and cereals or vegetables are among the first digestible dietary carbohydrates introduced at weaning (Stephen *et al*. 2012), the presence of such genetic elements for hydrolysis of glycosidic linkages may aid in their colonisation and persistence in the (infant) gut. Extensive research on carbohydrate utilisation in *B. breve* using a combination of comparative genomics, gene-trait matching (genotype-phenotype association) and functional genomics have also revealed that bifidobacterial mutualism and carbohydrate syntrophy occurs in the infant's gut wherein the species of *B. breve* may, perhaps, co-operatively, cross-feed with other (bifido)bacterial species like *B. bifidum* or *B. longum* subsp. *longum* in order to sustain growth on the various plant-derived carbohydrates (Pokusaeva, Fitzgerald and van Sinderen 2011; Egan *et al*. 2014b; Bottacini *et al*. 2018).

Furthermore, one study examining the metabolic capabilities of probiotic strains of bifidobacteria and lactic acid bacteria has revealed the superiority of HRB strains to assimilate galactooligosaccharide (GOS), a group of prebiotic compounds that are widely used in infant nutrition to stimulate growth of beneficial gut bacteria (Böger *et al*. 2019). HRB strains tested (*B. adolescentis* DSM 20 083, *B. breve* DSM 20 091, *B. bifidum* DSM 20 456, *B. longum* subsp. *infantis* DSM 20 088) grew well and were more efficient for GOS utilisation than non-HRB strains (*B. lactis* W51 and W52), with a distinct degree of polymerisation and different glycosidic linkage compositions. Noteworthy, most of the branched GOS were only consumed by HRB strains of *B. breve*, *B. adolescentis*, and *B. longum* subsp. *infantis* (Böger *et al*. 2019). This study suggests that HRB strains particularly *B. breve* and *B. longum* subsp. *infantis* are most suitable for symbiotic mixtures with GOS.

On the other hand, several species of non-HRB (*B. animalis* subsp. *lactis*, *B. pseudolongum* subsp. *pseudolongum*, *B. pseudolongum* subsp. *globusum*, and *B. thermophilum*) were found lacking the sets of genes encoding enzymes involved in the metabolism of arabinoxylan oligosaccharides (Rivière *et al*. 2014). Notably, the species of *B. animalis* subsp. *lactis –*a common probiotic agent used in dairy products and dietary supplements – can only metabolise a very minimal number of carbohydrates (Milani *et al*. 2013, 2015a). It is therefore plausible that the genetic architecture of non-HRB for plant-derived carbohydrates metabolism is associated with their corresponding hosts, revealing a co-evolution host–microbe profile. Nonetheless, such genetic elements may have undergone massive genome decay as a result of its industrial exploitation, which has involved long-term cultivation of *B. animalis* subsp. *lactis* on synthetic media (Milani *et al*. 2013).

HRB species have also been shown to be capable of utilising mucin as a nutrient source in order to colonise and survive within the host intestinal lining (Kiyohara *et al*. 2012; Katayama 2016). Mucins are host-derived glycans, secreted by intestinal goblet cells that essentially coat the surface of the intestinal mucosa. The main monosaccharides components are *N*-acetylglucosamine, *N*-acetylgalactosamine, and galactose, and these glycoproteins are decorated with fucose, sialic acid, and sulphate groups (Tailford *et al*. 2015). It has been reported that most strains of *B. longum* subsp. *longum* and *B. bifidum* are equipped with the gene homologs encoding endo-α-*N*-acetylgalactosaminidase and are thus capable of hydrolysing the core structures of mucin-type oligosaccharides, Galβ1–3GalNAcα-O-Ser/Thr (or T-antigen) to Galβ1–3GalNAc (Fujita *et al*. 2005). Also, infant-type HRB strains of *B. longum* subsp. *infantis*, *B. longum* subsp. *longum*, and *B. breve* have also been shown to possess the gene homolog encoding α-*N*-acetylgalactosaminidase (NagBb) that hydrolyses GalNAcα-O-Ser/Thr (Tn antigen) from the core-3-mucin-type *O*-glycans (GlcNAcβ1–3GalNAcα-O-Ser/Thr) (Odamaki *et al*. 2015). In particular, the strain *B. breve* UCC2003, which is a versatile bacterium from a metabolic perspective, was also shown to contain sulfatase-encoding gene clusters that allow it to metabolise *N*-acetylglucosamine-6-sulfate (GlcNAc-6-S), but apparently not on GlcNAc, as a sole carbon source (Pokusaeva, Fitzgerald and van Sinderen 2011; Egan *et al*. 2016). Interestingly, it is reported that *B. breve* UCC2003 produces an intracellular sulfatase enzymes and appears to employs a cross-feeding strategy with other members of the gut microbiota in order to gain access to mucin-derived sulphated monosaccharides (Egan *et al*. 2014a, 2016). Nonetheless, non-HRB strains of *B. animalis* were found lacking these genetic elements. These findings reflect the fitness and competitiveness of HRB strains, particularly the infant-type HRB, to survive and persist in the human intestine, where the glycans produced by the host serve as a carbon source for these bifidobacterial species.

Furthermore, these genetic data were substantiated by the analysis of mucin degradation of infant-type HRB, which highlighted the superior capability of the strains of *B. bifidum* D119 and L22, *B. breve* NCIMB8807 and *B. longum* subsp. *longum* NCIMB8809 to utilise mucin as a carbon source and grow well in mucin-containing defined medium (Ruas-Madiedo *et al*. 2008). In contrast, *B. animalis* and *B. pseudocatenulatum* strains, which are lacking mucin-utilisation-related genes (*afcA* and *engBF* glycosidase genes), were not able to utilise mucin and had minimal growth (Ruas-Madiedo *et al*. 2008). It is therefore implicated that HRB strains, particularly infant-type, are more competent to assimilate mucin, survive, and persist in the human gastrointestinal tract.

These findings suggest that HRB possess adaptive advantages and competitiveness to colonise in the human gut over the non-HRB that are less specialised with the human gut environment. Although the carbohydrates assimilation capability of bifidobacteria is more likely to be dependent on strain-specificity, the driving force of residential origin cannot be neglected. For instance, the HRB strains that are capable of utilising non-digestible glycans would aid in human digestion of glycans with valuable nutrient values which would otherwise be lost from the body and unused as waste to the outside environment.

### Production of folate

In addition to carbohydrate metabolisms and adaptation to a wide variety of carbohydrates, bifidobacterial species that are naturally residing in the human gut (HRB) also possess interesting feature concerning folate biosynthetic capabilities. Folate, also known as vitamin B9 or pteroyl-L-glutamate, is water-soluble vitamin that plays a role as a cofactor. It is required for efficient DNA replication, repair and methylation, and synthesis of nucleotides, vitamins and certain amino acids (Jacob 2000; Lucock 2000). For this key role in the cell cycle, various fast proliferating cells such as leukocytes, erythrocytes, and enterocytes require high levels of folate for growth (Jacob 2000).

Humans are auxotroph for folate; they cannot synthesise on their own and must obtain it exogenously through the diet (e.g. leafy green vegetables, yeast extracts, liver, beans) or indigenous folate-synthesising bacteria (Strozzi and Mogna 2008; Aufreiter *et al*. 2009). Folate deficiency has been associated with malformation of the neural tube during embryonic development and increased risk of megaloblastic anaemia, certain forms of cancer, and cardiovascular diseases (Rayburn, Stanley and Garrett 1996; Choi and Mason 2000; Stanger 2002). Moreover, this vitamin is particularly important for some population groups, including the elderly, children, and pregnant women.

The folate molecule contains a pterin moiety originating from 6-hydroxymethyl-7,8-dihydropterin pyrophosphate (DHPPP), bound to a unit of *para*-aminobenzoic acid (*p*ABA). The *de novo* biosynthesis process of folate necessitates both DHPPP and *p*ABA, for which the synthesis of these precursors requires the three basic building blocks: guanosine triphosphate (GTP), chorismate, and glutamate. DHPPP is formed from GTP in four consecutive steps, while *p*ABA can be produced from chorismate in the shikimate pathway (Rossi, Amaretti and Raimondi 2011; Andlid, D'Aimmo and Jastrebova 2018). Subsequently, the DHPPP and *p*ABA are joined together with the formation of a C-N bond, catalysed by the enzyme dihydropteroate synthase (DHPS, EC 2.5.1.15) to form 7,8-dihydropteroate (DHP). Further, DHP is reduced to the biologically active cofactor tetrahydrofolate (THF) (Rossi, Amaretti and Raimondi 2011; Andlid, D'Aimmo and Jastrebova 2018).

Bifidobacteria have been shown to possess the folate synthesising machinery, albeit not all species have the complete enzymatic armoury (Rossi, Amaretti and Raimondi 2011; LeBlanc *et al*. 2013). Comparative genomic analysis has provided intriguing insights into the genes and corresponding enzymes presumptively involved in bifidobacterial folate biosynthesis. Based on the available genome information, all sequenced bifidobacterial species have thus far shown to harbour the required genes for the shikimate pathway and thus can produce chorismate (Barrangou *et al*. 2009; Ventura *et al*. 2009; Turroni *et al*. 2010). However, not all species are capable of converting chorismate into *p*ABA. Although all species harbour *pabA* gene encoding ADCS enzyme, only the HRB species of *B. adolescentis* and *B. dentium* possess the *pabC* gene encoding for ADCL enzyme and can, therefore, synthesise *p*ABA *de novo* (Rossi, Amaretti and Raimondi 2011; Andlid, D'Aimmo and Jastrebova 2018). The other species would require *p*ABA supplementation in order to accomplish folate production.

Moreover, all sequenced HRB species (*B. adolescentis*, *B. dentium*, and *B. longum* subsp. *longum*) harbour a cluster of *fol* genes for DHPPP biosynthesis and are predicted to carry out the condensation of DHPPP and *p*ABA (Rossi, Amaretti and Raimondi 2011). The non-HRB species of *B. animalis* subsp. *lactis* were, however, found missing the key genes (*folE* and *folBK*) needed for DHPPP biosynthesis and the *folP* gene encoding DHPS, enzyme involves in the condensation of DHPPP and *p*ABA (Rossi, Amaretti and Raimondi 2011; LeBlanc *et al*. 2013; Milani *et al*. 2014), and are therefore auxotroph for folates even if *p*ABA is present in the environment. Collectively, it is conceivable that the HRB species of *B. adolescentis* and *B. dentium* can produce folate *de novo*, *B. longum* subsp. *longum* requires *p*ABA supplementation, and the non-HRB species of *B. animalis* requires folate supplementation.

Furthermore, many studies have assessed the capabilities of bifidobacterial species to produce folate and their possible contribution to the folate intake of the host (Pompei *et al*. 2007a; D'Aimmo et al. 2012, 2014; Sugahara *et al*. 2015). It appears that the mere ability to synthesise folate is restricted to certain species/strains of bifidobacteria, and to a large extent, their capabilities are also highly associated with the residential origins, leading to distinct differences between HRB and non-HRB in folate production (Sugahara *et al*. 2015). For instance, while most HRB strains were capable of producing high levels of folate, non-HRB strains produce the least amount or nothing (autotroph) (Sugahara *et al*. 2015). Interestingly, the folate production capacity in bifidobacteria is reported to be correlated with the phylogenetic lineage. Several HRB species isolated from non-human primates (*B. adolescentis* and *B. dentium*) were capable of producing a high level of total folate, whereas the non-HRB species isolated from non-primates (*B. animalis* subsp. *animalis*, *B. animalis* subsp. *lactis*, *B. pseudolongum* subsp. *globosum*, *B. asteroides*, *B. coryneforme*, and *B. indicum*) were incapable (D'Aimmo *et al*. 2014). It has become evident that folate production is characteristic of HRB but not of non-HRB. Adaptations to habitats that frequently contain external folate, mainly the animal intestines, have presumably led to reduced selection pressure for *de novo* folate biosynthesis and are most likely rendering many strains and possibly whole species of non-HRB to have lost their ability to synthesise folate (Andlid, D'Aimmo and Jastrebova 2018).

Some *in vivo* studies further confirmed the capabilities of HRB to produce high folate levels and to act as an endogenous source of this vitamin by elucidating its release inside the intestines of the murine model and human subject (Pompei *et al*. 2007b; Strozzi and Mogna 2008; Sugahara *et al*. 2015). In an *in vivo* study using germ-free mice administered with a single strain of bifidobacteria, the levels of folate were found to be higher in mice administered with the HRB strain of *B. longum* subsp. *longum* than the non-HRB strain of *B. animalis* subsp. *lactis*. This was accompanied by a significant improvement in the haematological indicators related to anaemia in germ-free mice colonised with HRB strain (Sugahara *et al*. 2015). The study suggests that folate-producing HRB strain could be more beneficial than those of the non-HRB strain in protecting the human host against folate deficiency health complications such as anaemia. Furthermore, in a human pilot study, daily consumption of the HRB strains (*B. adolescentis* DSMZ 18 352, *B. adolescentis* DSMZ 18 350, and *B. pseudocatenulatum* DSMZ 18 353) for 30 days resulted in a significant higher folate content in the intestinal lumen, indicating these HRB strains are capable of producing folate and providing an endogenous source of this vitamin to the human host (Strozzi and Mogna 2008).

The species of HRB have thus far been shown to harbour a functioning folate biosynthesis machinery and exhibit a greater capacity for folate production than the non-HRB. It seems possible that HRB species have been favoured during the evolution with the human host wherein they can both colonise the human gut and produce folate (symbiotic coevolution). Whether this is true or not is unknown but the findings show a great promise for high-folate producing HRB strains supplementation in improving human folate status. Folate-producing HRB strains, either incorporated in functional foods or as trophic probiotics, would help in intestinal homeostasis of vitamins and confer beneficial effects on human health.

### Degradation of food-derived opioid peptides

Food-derived opioid peptides are substances generated from enzymatic hydrolysis of dietary proteins, including milk, vegetable, cereal, and meat/poultry that are having morphine-like activities (Teschemacher 2003). Due to structural similarity to endogenous opioids, food-derived opioid peptides could be recognised by opioid receptors that are found primarily in the central nervous system and gastrointestinal tract, and display opioid-like molecular and physiological activities (Liu and Udenigwe 2019). The first identified and widely studied milk casein-derived opioid peptide was β-casomorphin-7 (BCM-7; Tyr-Pro-Phe-Pro-Gly-Pro-Ile) released from β-casein of bovine or human milk during technological processes and/or enzymatic digestion in the intestine (Brantl *et al*. 1981). Studies have suggested that BCM-7 may pass through human intestinal barriers, and could, in turn, influence on nervous, digestive and immune functions by altering the gene expression of µ-opioid receptors located on cell surfaces of these systems (Liu and Udenigwe 2019). BCMs -4, -5, and -6, which are derived by sequential removal of three, two or one amino acid residues, respectively from the C-terminus of BCM-7, have also been demonstrated to possess opioid agonist effects (Brantl *et al*. 1981). Also, gluten exorphin and gliadorphin-7 (or gliadinomorphin-7), derived from the gluten found in wheat flour, rye, barley and oats, were acknowledged as substances with opioid activity (Pruimboom and De Punder 2015).

Food-derived opioid peptides have received much attention in human health. Some food-derived opioid peptides have been reported to exert beneficial health bioactivities in healthy humans (Rutherfurd-Markwick 2012; Liu and Udenigwe 2019), however, some studies implicate that they may have adverse health effects in susceptible people. Milk β-casein-derived BCM-7, for instance, has been demonstrated to be associated with many negative health outcomes, including autism, type I diabetes, sudden infant death syndrome, and atopic dermatitis (Wasilewska *et al*. 2011; Fiedorowicz *et al*. 2014; Sokolov *et al*. 2014; Chia *et al*. 2017). BCMs have been reported to be a causal trigger to apnoea in sudden infant death syndrome for which penetration of BCMs into the infant's immature central nervous system may inhibit the respiratory centre in the brainstem, thereby leading to abnormal ventilator responses, apnoea, and death (Sun *et al*. 2003). Several other studies provide direct evidence that BCM-7 is positively correlated with childhood mental disorders and apparent-life threatening events in infants wherein an elevated level of BCM-7 was detected in the urine of autistic children (Sokolov *et al*. 2014) and the sera of infants with apnoea (Wasilewska *et al*. 2011). In addition, exclusion of gluten from the diet can restore regular gut mucosa functions and mitigate gastrointestinal inflammation (Jianqin *et al*. 2015) and celiac disease (Anania *et al*. 2017; Brietzke *et al*. 2018), both of which could lead to the development of other autoimmune disorders like type I diabetes (Serena *et al*. 2015; Krzewska and Ben-Skowronek 2016).

There is also evidence that patients who have celiac disease, an autoimmune disorder triggered by gluten ingestion in genetically predisposed individuals, had a decreased representation of bifidobacteria species in their gut microbiota as compared to those of the healthy subjects (Golfetto *et al*. 2014). Several attempts have been made to supplement some bifidobacterial strains as a probiotics therapy for celiac disease (Smecuol *et al*. 2013; Klemenak *et al*. 2015; Quagliariello *et al*. 2016; Pinto-Sánchez *et al*. 2017). For instance, in a double-blind, randomised, placebo-controlled trial, administration of *B. longum* subsp. *infantis* NLS-SS to celiac disease patients for three weeks resulted in symptomatic improvement (Smecuol *et al*. 2013). Similarly, administration of two probiotic strains, *B. breve* B632 and BR03, suppressed tumour necrosis factor-alpha (TNF-α) production in celiac disease children on a gluten-free diet and such positive effect was reversed after three months of trial where probiotics supplementation had ceased (Klemenak *et al*. 2015). Moreover, some bifidobacterial strains (*B. longum* subsp. *longum* IATA-ES1 and *B*. bifidum IATA-ES2) have also been shown to degrade gliadin peptides into inactive peptides, thereby reducing the cytotoxic and inflammatory effects of gluten peptides (Laparra and Sanz 2010; Cinova *et al*. 2011).

Noteworthy, many of the strains exerting positive effects on gluten-mediated disorders are the commensal bacteria of the human gut and belong to HRB, albeit some non-HRB strain (*B*. *animalis* subsp. *lactis*) was also reported to have a positive effect (Lindfors *et al*. 2008). It is therefore implicated that the capability to degrade the potentially harmful food-derived opioid peptides could be a specific physiological function of HRB, explaining at least in part why HRB naturally inhabit the human gastrointestinal tract across the lifespan and how they contribute to human health. Indeed, HRB species were found to possess more potent degradative capabilities for human milk- and bovine milk-derived BCM-7 as well as wheat gluten-derived α-gliadorphin-7 (Sakurai *et al*. 2018). Specifically, infant-type HRB strains were reported to exert a relatively higher dipeptidyl peptidase IV — a proline-specific enzyme that can hydrolyse opioid peptides — activity than the other strains wherein certain strains of *B. longum* subsp. *infantis* and *B. bifidum* demonstrated the greatest degradative capabilities for all three food-derived opioid peptides. In contrast, the strains of non-HRB were inactive for the hydrolysis of food-derived opioid peptides (Sakurai *et al*. 2018). Taken together, these findings provide some clues on the possible role of HRB as prominent members of the human gut microbiota. It is implicated that HRB species could aid in the degradation of the potentially harmful food-derived opioid peptides, particularly the strains of infant-type HRB as of which these peptides are more potent to infants with immature gastrointestinal system, thereby contributing to host health.

### Production of aromatic lactic acids

Another vital feature of HRB species is their capability to produce tryptophan-derived indoles. Gut bacterial tryptophan metabolites have increasingly been recognised as an essential mediator in host physiology and may contribute to intestinal and systemic homeostasis (Tremaroli and Bäckhed 2012; Agus, Planchais and Sokol 2018). For instance, microbial tryptophan-derived indoles, including indole-3-acetic acid (IAA), indole-3-aldehyde (IAld), indole-3-lactic acid (ILA) and indole-3-propionic acid (IPA), have been reported to act as ligands of aryl hydrocarbon receptor (AhR) and may potentially improve gut barrier function and regulate gut mucosal immune responses (Hubbard *et al*. 2015; Cervantes-Barragan *et al*. 2017; Dodd *et al*. 2017; Wilck *et al*. 2017). ILA and IPA have also been identified as antioxidants and as free-radical scavengers (Chyan *et al*. 1999; Karbownik *et al*. 2006; Suzuki *et al*. 2013). In this regard, it seems likely that the production of tryptophan-derived indoles could be the precise mechanism deploys by HRB species in host-microbial interactions, contributing to their beneficial effects on human health.

This concept has been demonstrated in a recent study investigating the anti-inflammatory effect of ILA — a breastmilk tryptophan metabolite secreted by *B. longum* subsp. *infantis* — in the immature intestine (Meng *et al*. 2020). The study found that this tryptophan metabolite produced by an infant-type HRB strain *B. longum* subsp. *infantis* ATCC 15 697 interacts with AhR and reduces the inflammatory interleukin-8 (IL-8) response in immature but not mature intestinal enterocytes (Meng *et al*. 2020). Studies have shown that ILA is involved in host immune homeostasis via AhR activation, leading to reprogramming of immunoregulatory T cells and inflammatory T cells (Cervantes-Barragan *et al*. 2017; Lanz *et al*. 2017; Wilck *et al*. 2017). Such function would be beneficial for healthy growth, including the immune development and maturation, in infants.

Interestingly, in another recent report, ILA was found as the only tryptophan metabolite produced by bifidobacterial species; no others, including IPA, IAA, and IAld, were produced (Sakurai, Odamaki and Xiao 2019). Noteworthy, the species of infant-type HRB displayed the highest capacity for ILA production among which the strains of *B. longum* subsp. *longum*, *B. longum* subsp. *infantis*, *B. breve*, and *B. bifidum*, produced higher levels of ILA than other strains (Sakurai, Odamaki and Xiao 2019). Numerous studies have also demonstrated that infant-type HRB are particularly superior in producing ILA (Aragozzini *et al*. 1979; Russell *et al*. 2013; Ehrlich *et al*. 2018). Nevertheless, the exact biological role of ILA; how and why only limited species (infant-type HRB) are allowed to harbour in the human infant gut, remains unclear. Very recently, in a preprints, Laursen *et al*. 2020 demonstrated that aromatic lactate dehydrogenase, an enzyme which was explicitly found in breastmilk-promoted *Bifidobacterium* species (infant-type HRB), is involved in the formation of aromatic lactic acid metabolites including ILA from aromatic amino acids. They also showed that stool concentrations of aromatic lactic acids are determined by the abundance of breast milk-promoted *Bifidobacterium* species harbouring the aromatic lactate dehydrogenase enzyme. ILA was pointed as a relevant early life AhR agonist and could be associated with intestinal and systemic homeostasis. ILA has been reported to have antimicrobial activity (Shigeno *et al*. 2018) in addition to hydrogen peroxide production as a by-product during tryptophan deamination (Dieuleveux, Lemarinier and Gueguen 1998), suggesting ILA production by infant-type HRB may contribute to their preponderance and competitiveness in the infant intestines. It is therefore implicated that bifidobacteria-derived ILA may have conserved roles in human health development. Taken together, these findings shed lights on the mechanism by which HRB, especially the infant-type, exert their adaptive abilities and beneficial properties in the human gut.

## CONCLUDING REMARKS

Through more than a century after its discovery, evidence has indicated that bifidobacteria have coevolved with their respective hosts. Conversely, bifidobacterial species that naturally occur in the human gut, better known as HRB, have adapted to the human host and possess many unique physiological characteristics. This organism is seen as an essential member of the human gut microbiota across the lifespan, and is associated with human health. The acquisition of HRB species commences from birth, for which the bacteria are vertically transmitted from the mother's vaginal tract, gastrointestinal tract, breast milk, placenta and amniotic fluid to the infant. The studies, to date, have revealed that the persistence of infant-type HRB in the infant gut is attributed to their competitive abilities with regards to human milk (i.e. their ability to metabolise HMOs and tolerance to lysozyme). Noteworthy, infant-type HRB are naturally selected by human milk; they are genetically equipped and adapted to assimilate HMOs, explaining why the species of infant-type HRB have such a close relationship to the breast-fed infant. With a progressive reduction of breastfeeding and an increase of solid food intake, the species of HRB are then shifted from infant-type to adult-type. During adulthood, although their abundance is relatively low, HRB may still impact host overall health through their metabolic and physiological activities. In particular, adult-type HRB appear to possess a large amount of the gut-associated enzymatic arsenal dedicated to the metabolism of dietary plant polysaccharides and host-derived carbohydrates, thus enforcing the importance of HRB in facilitating the metabolism of complex carbohydrates that are not digested by human intestinal enzymes or by other gut bacteria. Besides, it has become clear that certain strains of HRB, particularly infant-type HRB, have a better functional capacity in various physiological activities, including the production of folate and ILA as well as degradation of food-derived opioid peptides. The studies discussed here shed lights on the role of HRB, particularly infant-type HRB in most of the cases, as members of human gut microbiota across the lifespan. Despite such findings, many potential physiological characteristics of HRB have yet to be explored. Furthermore, we still lack a comprehensive understanding of how HRB species interact with the human host and contribute to human health. More studies are thus needed to provide in-depth insight into the evolutionary scenario of this critical human gut commensal bacteria, which would, in turn, facilitate the selection of strains for application as human probiotics.
